# Adjuvant radiotherapy of regional lymph nodes in breast cancer - a meta-analysis of randomized trials

**DOI:** 10.1186/1748-717X-8-267

**Published:** 2013-11-14

**Authors:** Wilfried Budach, Kai Kammers, Edwin Boelke, Christiane Matuschek

**Affiliations:** 1 Medical Faculty, Department of Radiation Oncology, Heinrich Heine University of Düsseldorf, Moorenstraße 5, Düsseldorf, D-40225, Germany; 2 Department of Biostatistics, John Hopkins Bloomberg School of Public Health, Baltimore, MD, USA

**Keywords:** Meta-analysis, Breast cancer, Radiotherapy, Lymph node, Internal mammary, Medial supraclavicular

## Abstract

**Background:**

Radiotherapy (RT) improves overall survival (OS) of breast cancer patients after breast conserving surgery and after mastectomy in patients with involved lymph nodes (LN). The contribution of RT to the regional LN to this survival benefit was poorly understood. Recently, the results of three large randomized trials addressing this question have become available.

**Material and methods:**

The published abstracts (full publication pending) of the MA.20 (n=1832) and the EORTC 22922–10925 (EORTC) (n=4004) trial and the full publication of the French trial (n=1334) were basis of the meta-analysis. Main eligibility criteria were positive axillary LN (all trials), LN negative disease with high risk for recurrence (MA.20), and medial/central tumor location (French, EORTC). The MA.20 and the EORTC trial tested the effect of additional regional RT to the internal mammary (IM) LN and medial supraclavicular (MS) LN, whereas in the French trial all patients received RT to the MS-LN and solely RT to the IM-LN was randomized. Primary endpoint was OS. Secondary endpoints were disease-free survival (DFS) and distant metastasis free survival (DMFS).

**Results:**

Regional RT of the MS-LN and the IM-LN (MA.20 and EORTC) resulted in a significant improvement of OS (Hazard Ratio (HR) 0.85 (95% CL 0.75 - 0.96)). Adding the results of the French trial and using the random effects model to respect the different design of the French trial, the effect on OS of regional radiotherapy was still significant (HR 0.88 (95% CL 0.80 - 0.97)). The absolute benefits in OS were 1.6% in the MA.20 trial at 5 years, 1.6% in the EORTC trial at 10 years, and 3.3% in the French trial at 10 years (not significant in single trials). Regional radiotherapy of the MS-LN and the IM-LN (MA.20 and EORTC) was associated with a significant improvement of DFS (HR 0.85 (95% CL 0.77 - 0.94)) and DMFS (HR 0.82 (95% CL 0.73 - 0.92)). The effect sizes were not significantly different between trials for any end point.

**Conclusion:**

Additional regional radiotherapy to the internal mammary and medial supraclavicular lymph nodes statistically significantly improves DFS, DMFS, and overall survival in stage I-III breast cancer.

## Introduction

Clinical data indicate that breast cancer is a radiosensitive disease. Adjuvant radiotherapy after breast-conserving surgery reduces the risk of ipsilateral in breast recurrence by at least a factor of 3 and halves the risk of any disease recurrence resulting in a significantly improved overall survival [1,2]. Radiotherapy after mastectomy in node positive breast cancer patients reduces chest wall recurrences by a factor 3–4 and improves overall survival by 6% [3]. Whereas almost all patients in clinical trials on postmastectomy radiotherapy received comprehensive nodal irradiation including the axillary, medial supraclavicular (MS), and internal mammary (IM) lymph nodes, the majority of patients in trials on radiotherapy after breast conserving surgery did not receive nodal radiotherapy except in some trials for node positive disease. Whether and to which extent nodal radiotherapy contributed to improved disease free and overall survival in these trials is unknown. Since surgical treatment of the axilla in clinical node negative breast cancer has not consistently shown to improve overall survival, the less aggressive sentinel node biopsy (SNB) technique is considered as standard treatment. The slightly higher axillary recurrence rate after SNB compared to axillary dissection was shown to have no impact on overall survival [4] supporting the SNB concept and indirectly the hypothesis that involved LN in breast cancer indicate a high risk of systemic disease, but do not represent a major source of systemic disease [5]. The work published by Veronesi and colleagues [6] that was done before any systemic treatment of breast cancer had been established, can be interpreted in the same direction. In their randomized trial (n = 737) that tested the resection of IM-LN in addition to mastectomy and axillary surgery, no survival benefit of the extended surgical approach was shown in spite of the fact that pathological involvement of the internal mammary LN was confirmed in 21% of patients.

In non-randomized comparisons, radiotherapy to the internal mammary lymph nodes (IM-LN) was reported either to be associated with a significantly improved [7-9] or a trend to an improved survival [10,11], or no improvement of survival [12].

Elective radiotherapy to the medial supraclavicular LN reduced the supra/infraclavicular relapse rate at 10 years from 4.2% to 1% in a randomized Danish trial [13] on postmastectomy radiotherapy in postmenopausal patients. Yates and colleagues [14] reported a 30% supraclavicular relapse rate at 10 years without radiotherapy in high risk patients, defined as G3 tumors with >2 involved axillary LN. The potential reduction of nodal recurrence by regional radiotherapy may be in the same order of magnitude for the supraclavicular compared to the internal mammary LN. However, no relevant data on the effect elective radiotherapy to the supraclavicular LN on survival are available.

The first results of randomized trials addressing the questions have recently been published or were presented on scientific meetings. The potentially important implications for the management of breast cancer prompted us to perform an early meta-analysis based on already available information.

## Material and methods

**Figure 1:**
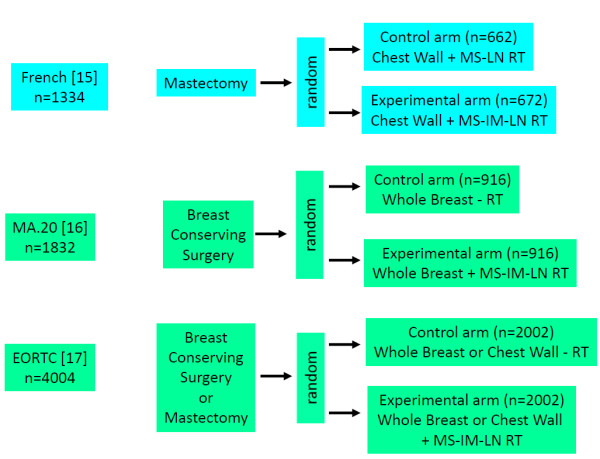
**Trial designs.** Random = randomization. RT = radiotherapy. MS-LN-RT = radiotherapy of medial supraclavicular lymph nodes. MS-IM-RT = radiotherapy of medial supraclavicular and internal mammary lymph nodes.

Using the search term “breast cancer” and radiotherapy and (regional or nodal or “internal mammary” or parasternal or supraclavicular) restricted to “randomized controlled trial” or “clinical trial, Phase III” in Pubmed (September 2013) yielded 150 publications of potential interest. In none of these publications, results of a randomized comparison of regional radiotherapy to the internal mammary nodes and/or to the supraclavicular nodes versus no radiotherapy were reported. Interestingly, the recently published results from a French trial [15] was not identified, since it was categorized as “clinical trial” and not as “randomized controlled trial” in Pubmed. In addition, abstracts published between 2008 to September 2013 of important annual cancer meetings were screened (ASCO, ASTRO, ECC, ESTRO, San Antonio Breast Cancer Meeting). Two trials of interest that have so far been published in abstract form only, the MA.20 [16] and the EORTC 22922–10925 [17] were additionally identified. In total, information from three randomized trials comparing regional radiotherapy to regional radiotherapy was available (Table 1). Two trials ([16,17]) tested the effect of additional IM-LN and MS-LN radiotherapy and one trial [15] the effect of additional radiotherapy of the IM LN (Figure 1). Details of the radiation techniques used in the EORTC 22922–10925 and the French trial have been published [14,18]. In the MA.20 trial, MC LN and level 3 axillary LN were treated with an anterior filed. For radiotherapy of the IM LN a modified wide tangent technique or a direct field matched to tangent fields were used. The inclusion criteria of the trials were similar but not identical (Table 1). The majority of patients had node positive disease or medial/central tumors and received systemic chemotherapy. The primary end point of all trials was overall survival. Secondary endpoints were disease-free survival (DFS), distant metastasis free survival (DMFS) and locoregional tumor control. Data on DFS and DMFS survival were available only from the MA.20 and the EORTC 22922–10925, but not from the French trial. Since information on regional tumor control was available from the MA.20 only, this endpoint was not included in the meta-analysis.

**Table 1:** Patient characteristic

	**MA.20**[16]	**EORTC**[17]	**French**[15]
**Recruitment years**	2000-2007	1996-2004	1991-1997
**Number of patients**	1832	4004	1334
**Median age**	54	54	57
**Node positive**	85%	56%	75%
**Breast surgery**	100% breast conserving	75% breast conserving	100% mastectomy
**CHX**	91%	85%	61%
**ER/PR negative**	25%	16%	7%
**Unknown ER/PR status**	n.a.	6%	40%
**Main inclusion criteria**	N + or high risk* N0 any location	N + or medial/central tumor	N + or medial/central tumor
**Breast/chest wall**	Both arms: 50 Gy / 25 fx	Both arms 50 Gy / 25 fx	Both arms according to practice of the center
**Medial supraclavicular nodes**	Experimental arm: 45 Gy / 25 fx	50 Gy / 25 fx	All patients: dose and fractionation according to practice of the center
**Internal mammary nodes**	Experimental arm: 45 Gy / 25 fx	Experimental arm: 50 Gy / 25 fx	Experimental arm: 45 Gy / 20 fx

*= > = 5 cm tumor, > = 2 cm tumor, and <10 axillary nodes removed with ER-, G3, or lymph vacular invation; n.a. = not available; fx = fractions; ER = estrogene receptor; PR = progesterone receptor.

### Statistical analysis

All analyses were stratified by trial. For analysis, hazard ratios with 95% confidence limits for overall survival, DFS, and DMFS were derived from the abstracts of the MA.20 and the EORTC 22922–10925: The hazard ratio for overall survival of the French trial was derived from published survival curves according to the method described by Parmar et al. [19], since no information on hazard ratios were given in the publication. Taking into account the detailed information on the patients at risk during follow up, this method is able to estimate the hazard ratio quite accurately. Meta-analyses of the effect sizes of the MA.20 and the EORTC 22922–10925 trials on overall survival, DFS and DMFS were performed using fixed effect model based on parameter estimates of log hazard ratios in Cox models and their standard errors. Since the design of the French trial was different from the MA.20 and the EORTC 22922–10925 trials, the combined effect size for overall survival of all three trials were calculated based on the random effects model. Results are presented with forest plots, in which the estimates of the hazard ratios of all single studies and their combined estimate are visualized. Horizontal bars indicate the amount of variation (95% confidence intervals of the parameter estimates).

## Results

**Figure 2:**
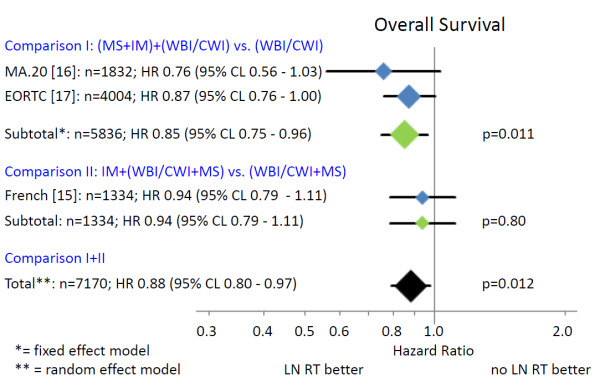
**Overall survival.** The area of the symbols reflect the number of patients, MS + IM = medial supraclavicular and internal mammary lymph node irradiation, WBI/CWI = whole breast irradiation or chest wall irradiation, MS = medial supraclavicular lymph node irradiation.

A total of 7170 breast cancer patients from three randomized trials were finally identified for the meta-analysis. Patients’ characteristics in the different trials are shown in Table 1. Comprehensive regional radiotherapy (Figure 2) of the MS-LN and the IM-LN (MA.20 and EORTC 22922–10925) resulted in a statistically significant improvement of overall survival (Hazard Ratio (HR) 0.85 (95% CL 0.75 - 0.96)). A small, but statistically not significant, improvement in overall survival was detected in the French trial, in which all patients received MS-RT and only the effect of additional radiotherapy to the IM-LN was tested (HR 0.94 (95% CL 0.79 - 1.11)). Adding the results of the French trial to the results of other trials (Figure 2) and using the random effects model to take into consideration that the design of the French trial was not identical, the effect on overall survival of regional radiotherapy was still statistically significant (HR 0.88 (95% CL 0.80 - 0.97)). The absolute benefits in overall survival were 1.6% in the MA.20 trial at 5 years, 1.6% in the EORTC 22922–10925 trial at 10 years, and 3.3% in the French trial 10 years.

**Figure 3:**
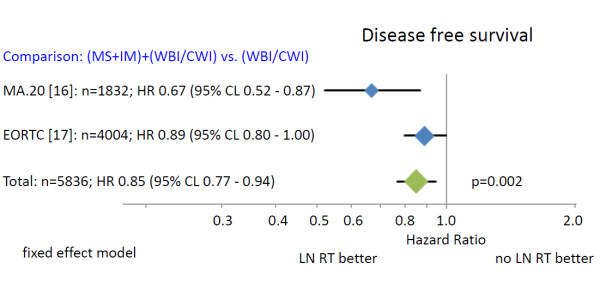
**Disease free survival.** The area of the symbols reflect the number of patients. MS + IM = medial supraclavicular and internal mammary lymph node irradiation, WBI/CWI = whole breast irradiation or chest wall irradiation.

Regional radiotherapy (Figure 3) of the MS-LN and the IM-LN (MA.20 and EORTC 22922–10925) was associated with a statistically significant improvement of DFS (HR 0.85 (95% CL 0.77 - 0.94)). The absolute benefits in DFS were 5.7% in the MA.20 trial at 5 years and 3.0% in the EORTC 22922–10925 trial at 10 years. Since no information on DFS was available from the French trial, a combined analysis was not possible.

**Figure 4:**
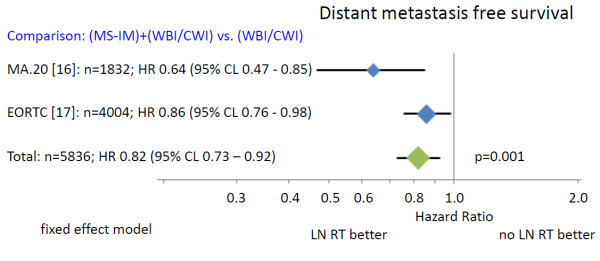
**Distant metastasis free survival.** The area of the symbols reflect the number of patients, MS + IM = medial supraclavicular and internal mammary lymph node irradiation, WBI/CWI = whole breast irradiation or chest wall irradiation.

Regional radiotherapy (Figure 4) of the MS-LN and the IM-LN (MA.20 and EORTC 22922–10925) significantly improved DMFS (HR 0.82 (95% CL 0.73 - 0.92)). The absolute benefits in DMFS was 5.4% in the MA.20 trial at 5 years and 3.0% in the EORTC 22922–10925 trial at 10 years. Again, no information of DMFS was available from the French trial, precluding a combined analysis.

The effect sizes were not significantly different between trials for any end point.

## Discussion

The combined results of randomized trials indicate a statistically significant improvement in overall survival for regional nodal radiotherapy in stage I-III breast cancer patients (Figure 2). The absolute overall survival benefit at 5 and 10 years was relatively small (1.6% to 3.3%), but the available survival curves from the MA.20 trial indicate that a larger benefit may occur with longer follow up. This interpretation is supported by the considerably larger effect of regional radiotherapy on DFS and DMFS (Figures 3 and 4). Surprisingly, the advantage in overall survival appears to be a consequence of the reduction of distant metastasis rate rather than improved regional tumor control as indicated by the almost identical effect of regional radiotherapy on DFS and DMFS. Data on regional tumor control is currently available only from the MA.20 trial. Regional radiotherapy improved regional tumor control at 5 years by 2.3%, but reduced the distant metastasis rate at 5 years by 5.4%. The question is whether this effect is real or an artefact. A possible explanation could be a considerable underestimation of the recurrence rate in the IM-LN. Recurrences in this nodal region are not detected by routine follow up programs. FDG-PET-CT scans at the time of the clinical appearance of distant metastasis have shown high rates of unsuspected mediastinal lymph node involvement [20] that may have originated in the IM-LN as source of further dissemination. Another hypothesis compatible with the observations is that micro-metastasis in the IM-LN and the MS-LN represent a source for metastatic spread without growing to clinically detectable size before distant metastases have been diagnosed. Both explanations would confute the hypothesis that regional lymph nodes in breast cancer do not represent a major source of distant disease [5]. The so called abscopal effect of radiotherapy, which is caused by an immune reaction against the tumor induced by tumor cell necrosis or necroptosis after ionizing radiotherapy [21,22] could also serve to explain the observations. This would, however, implicate that a relatively small number of tumor cells in clinically negative LN would be able to initiate a substantial immune response, which appears to be unlikely in view of no clinical evidence for such a reaction after radiotherapy of macroscopic disease in breast cancer. Which hypothesis is correct or whether combined effects or a completely different mechanism prevails, will be the subject of future investigations.

In view of the relatively small average survival advantage, implementation of regional radiotherapy into routine practice will foreseeable cause a lot of discussion in the scientific community. A meta-analysis on the individual patient’s data would likely be very helpful in this regard by identifying subgroups of patients with larger and smaller benefits than the average effect size. This is one important limitations of the current meta-analysis besides the fact that it is based on the restricted information provided in the abstracts and presentations in 2 out of 3 trials. A meta-analysis of specific subgroup was not possible, because information on subgroups from the different trials were available for incomparable subgroups only. In the French trial a trend for an improved overall survival was observed for the addition of radiotherapy to the IM-LN for patients with involved axillary nodes regardless whether patients received adjuvant chemotherapy or tumors were located in the central and medial quadrants or in the lateral quadrants of the breast. For patients without involved axillary LN a trend to a decreased overall survival was seen for IM-LN radiotherapy. In a subgroup analysis given in the oral presentation of the EORTC 22922–10925 trial on the ECCO-2013 congress (data not given in the abstract), patients, who had received both, adjuvant chemotherapy and endocrine treatment, had the largest survival advantage from regional lymph node radiotherapy (HR 0.72 (95% CL 0.55-0.94)). According to the treatment standards at the time of patient’s accrual, the majority of patients in this subgroup probably had node positive or high risk node negative disease and positive hormone receptor status. Anthracycline based adjuvant chemotherapies were already used in the MA.20 trial and in many patients in the EORTC trial. However, other modern adjuvant systemic treatments containing taxanes or trastuzumab had not been established at the time, when these trials were designed and were accordingly not used in the trials. There no doubt that these treatments would further improve clinical outcome [23,24] and would lower the total number of events. According to pervious experiences on the combination of systemic treatments with radiotherapy in breast cancer [1], one would expect that the hazard ratios for DFS and DMFS for regional radiotherapy remain constant, but the absolute benefits would be lower. Since the effect of additional adjuvant taxane treatment is relatively small (Relative risk for recurrence 0.84 - 0.86); [24], one would still expect a relevant benefit from regional radiotherapy. The effect of adjuvant trastuzumab in Her2 positive breast cancer [23] is considerably larger (odds ratio for recurrence: 0.53). Accordingly, the absolute benefit of regional radiotherapy may clinically be less relevant. While these considerations remain somewhat speculative, one has also keep in mind that a repeat of these large clinical trials with modern systemic treatments would take at least another decade and is unlikely to be performed.

The designs of the EORTC 22922–10925 and the MA.20 trials do not allow to answer the question whether both nodal regions need to be irradiated to achieve the observed survival advantage or radiotherapy of one nodal region (IM-LN or MS-LN) would be sufficient. In the French trial all patients received radiotherapy of the MS-LN and only radiotherapy of the IM-LN was subject of randomization. If radiotherapy of both nodal areas would be of equal importance, the effect size in the French trial should be half of the effect in the two other trials, which is approximately in accordance with the observations (Figure 2). However, one can also find arguments to claim that the predominant effect comes from MS-LN radiotherapy or IM-LN radiotherapy.

Radiotherapy of the IM-LN undoubtedly increases the dose to the heart regardless of the employed radiation technique. However, modern radiation technology keeps the dose to the heart considerably lower than formerly possible [25,26]. Almost all evidence for an access of cardiac death associated with radiotherapy in breast cancer, even in a recently published work [27], comes from patients that were treated with outdated radiation technology. Radiotherapy of the IM-LN was not associated with an excess of cardiac death or cardiac toxicity rate in any of the three trials discussed in this meta-analysis (Table 2). While the median follow up of the MA.20 trial (62 months) has to be regarded as insufficient to exclude relevant late cardiac toxicity, the median follow up of the EORTC 22922–10925 trial (10.9 years) and French trial (11.3 years) is already long enough to conclude that even with the radiation technique use in the 90ies, cardiac toxicity remains probably low. Advanced radiation technology that is now available in most centers, further decrease the dose to the heart [28]. The fear of late cardiac toxicity does not longer seem to be a relevant argument against radiotherapy to the IM-LN with the possible exception of patients, who receive trastuzumab or other anti-Her2-targeting drugs, since long term follow up in combination with radiotherapy to the IM-LN is still missing.

**Table 2:** Late toxicity in breast cancer trials on regional radiotherapy

**Trial late toxicity**	**MA.20**[16]			**EORTC**[17]			**French**[15]		
	**MS-IM-**	**IM-IM+**	**p**	**MS-IM-**	**IM-IM+**	**p**	**MS+**	**IM-IM+**	**p**
**Lung**									
Grade 2	0.2%	1.3	0.01	n.a.	n.a.	n.a.	n.a.	n.a.	n.a.
Grade >2	0%	0%	n.a.	n.s.					
Any grade n.a	n.a.	n.a.	n.a.	1.3%	4.3%	<0.0001	n.a.	n.a.	n.a.
**Lymphedema**									
Grade 2	3.7%	6.8%	0.004	n.a.	n.a.	n.a.	n.a.	n.a.	n.a.
Grade >2	0.4%	0.4%	n.s.	n.a.	n.a.	n.a.	n.a.	n.a.	n.a.
Any grade (arm)	n.a.	n.a.	n.a.	3.6%	3.8%	n.s.	n.a.	n.a.	n.a.
**Cardiac**									
Any grade	n.a.	n.a.	n.a.	1.4%	1.6%	n.s.	1.7%	2.2%	n.s
**Total late**									
Any grade	n.a.	n.a.	n.a.	21.8%	25.5%	0.006	n.a.	n.a.	n.a.
Grade >2	n.a.	n.a.	n.a.	n.a.	n.a.	n.a.	2.3%	3.1%	n.s.

Late toxicitiy.

n.a. = not available; n.s. = not significant; MS-IM-: no radiotherapy of the medial supraclavicular and internal mammary lymph nodes; MS-IM+: radiotherapy of the medial supraclavicular and internal mammary lymph nodes; MS+: radiotherapy of the medial supraclavicular lymph nodes.

Information regarding other important toxicities is available from the French trial (LIT) and the EORTC 22922–10925 [29] as full publications and from the MA.20 trial in abstract form (Table 2). The addition of radiotherapy to the IM-LN and MS-LN in the MA.20 and the EORTC 22922–10925 trial resulted in a statistically significant 1-3% increase in grade 1–2 lung toxicity. A 3% increase in grade 2 lymphedema was observed in the MA20 trial, but no increase was reported from EORTC 22922–10925 trial. The addition of radiotherapy to the IM-LN did not result in higher rates of acute or late toxicities in French trial (all patients received MS-LN radiotherapy), indirectly indicating that the moderately enhanced toxicities in the MA.20 trial and the EORTC trial could be predominantly associated with MS-LN radiotherapy rather than with IM-LN radiotherapy. The slightly higher lung toxicity that has not been reported to be more serve than grade 2 and a possibly higher likelihood of grade 2 lymphedema, should, at least to the opinion of the authors, not serve as strong argument against regional LN radiotherapy in view of a significant survival benefit. In clinical practice, risks and benefits need to be taken into consideration on an individual basis. Recommendations in treatment guidelines will require the information given in the pending full publications of the MA.10 and EORTC trial.

## Conclusion

Additional regional radiotherapy to the internal mammary and medial supraclavicular lymph nodes statistically significantly improves DFS, DMFS, and overall survival in stage I-III breast cancer.

## Competing interests

WB has no conflict of interests. KK has no conflict of interests. EB received travel grants form Dr. Sennewald Medizintechnik GmbH. CM received travel grants from Dr. Sennewald Medizintechnik GmbH.

## Authors’ contributions

WB had the idea and wrote most parts of the manuscript. KK did the statistical analysis and wrote the section “statistical analysis”. EB prepared figures and tables and contributed to the writing of the manuscript. CM did the systematic literature research and prepared the data for statistical analysis. All authors read and approved the final manuscript.
